# P-Glycoprotein and Drug Resistance in Systemic Autoimmune Diseases

**DOI:** 10.3390/ijms15034965

**Published:** 2014-03-20

**Authors:** Andrea Picchianti-Diamanti, Maria Manuela Rosado, Marco Scarsella, Bruno Laganà, Raffaele D’Amelio

**Affiliations:** 1 Department of Medical Sciences, Sapienza University of Rome, 2nd School of Medicine, Sant’ Andrea University Hospital, 00189 Rome, Italy; E-Mails: blagana@infinito.it (B.L.); raffaele.damelio@uniroma1.it (R.D.A.); 2 Research Center Ospedale Pediatrico Bambino Gesù, IRCSS, Piazza S. Onofrio 4, 00165 Roma, Italy; E-Mails: manuelaanjosm.rosado@gmail.com (M.M.R.); marco@libero.it (M.S.)

**Keywords:** p-glycoprotein, autoimmune diseases, multidrug resistance, lymphocytes

## Abstract

Autoimmune diseases such as systemic lupus erythematosus (SLE), rheumatoid arthritis (RA) and psoriatic arthritis (PsA) are chronic inflammatory disorders of unknown etiology characterized by a wide range of abnormalities of the immune system that may compromise the function of several organs, such as kidney, heart, joints, brain and skin. Corticosteroids (CCS), synthetic and biologic immunosuppressive agents have demonstrated the capacity to improve the course of autoimmune diseases. However, a significant number of patients do not respond or develop resistance to these therapies over time. P-glycoprotein (P-gp) is a transmembrane protein that pumps several drugs out of the cell, including CCS and immunosuppressants; thus, its over-expression or hyper-function has been proposed as a possible mechanism of drug resistance in patients with autoimmune disorders. Recently, different authors have demonstrated that P-gp inhibitors, such as cyclosporine A (CsA) and its analogue Tacrolimus, are able to reduce P-gp expression and or function in SLE, RA and PsA patients. These observations suggest that P-gp antagonists could be adopted to revert drug resistance and improve disease outcome. The complex inter-relationship among drug resistance, P-gp expression and autoimmunity still remains elusive.

## 1. Introduction

In the last decade, the growing knowledge of the molecular and cellular pathways involved in the development and progression of systemic autoimmune disorders combined with the advances in monoclonal antibody technology [1] have led to a better therapeutic management of autoimmune diseases. Despite these efforts, a significant percentage of patients do not respond, or develop a secondary failure to conventional treatment regimens. It has been hypothesized that therapeutic unresponsiveness may be caused by various mechanisms such as the rapid degradation of corticosteroids (CCS), the release of neutralizing antibodies against biological agents such as infliximab, or other unidentified factors [2].

More recently, unresponsiveness to CCS has been correlated to the expression of multidrug resistance gene (*MDR-1*) on a subset Th17 inflammatory lymphocytes [3]. *MDR-1* encodes for a transmembrane P-glycoprotein (P-gp), of 170-kD belonging to the superfamily of ABC (ATP binding cassette) transporters [4] that plays an important role in controlling drug uptake and excretion [5]. Initially studied in the context of tumor therapy, P-gp over-expression or hyper-function has been proposed, more recently, as a possible mechanism of drug resistance in patients with systemic autoimmune diseases [6,7].

In this review we will focus on the role of P-gp expression/function in the development of drug resistance in patients affected by systemic autoimmune diseases in particular systemic lupus erythematosus (SLE), rheumatoid arthritis (RA) and psoriatic arthritis (PsA) and will discuss how P-gp may be a therapeutical target in the control of abnormal immune response and inflammation.

## 2. P-gp Expression and Function in the Immune System

At least 48 human ABC transporters have been described, however only three have been linked to a role in mutidrug drug resistance (MDR) to anti-cancer, anti-inflammatory and anti-viral drugs [8]: the multidrug resistance associated protein 1 (MRP1 or ABCC1), the breast cancer resistance protein (BCRP or ABCG2) and P-gp also called transmembrane small-molecule pump (ABCB1). P-gp is one of the most studied MDR family members for its function in extruding various cytotoxic compounds out of the cells [9] but also for its role in modulating inflammation by direct or indirect tuning the secretion of cytokines, chemokines and other small peptides [10–12].

P-gp is widely present in different normal tissues such epithelial cells of the kidney, liver, intestine and in endothelial cell of the brain and of the placenta [13,14]. P-gp is also present at different stages of the lymphoid cell development [15–17] but its role on the maturation and function of each cell subset has not been completely revealed. Recently, studies in the mouse have shown that P-gp expression is required for dendritic cell (DCs) migration to lymph nodes [18] as well as for DCs development and maturation [19]. In fact, the down-modulation of P-gp on DCs, after venlafaxine (VLX) treatment, dampens surface expression of co-stimulatory molecules and reduces cytokine production impairing T cell proliferation in an allogenic mixed lymphocyte reaction (MLR) assay. In mice, the ablation of the *MDR-1a* gene [20,21], that codes for P-gp, always leads to the spontaneous development of T-cell mediated colitis with no other autoimmune disorder being reported [22,23]. Recently this mouse model for colitis has been the target of a new study in which the role of P-gp expression and the homeostasis of the regulatory T cell compartment was investigated [24–27]. It was found that P-gp is important for the generation, at the mucosal site, of inducible regulatory T cells (iTreg) from naïve *MDR-1a* deficient T cells. Thus, lack of P-gp on CD4+ T cells compromises the suppressive function and the anti-inflammatory role played by iTreg cells in the intestine finally resulting in the development of chronic inflammation and colitis [28].

As in the mouse, in humans, the expression of P-gp in the T cell compartment seems to be tightly regulated. P-gp is highly expressed by bone marrow multipotent stem cells in humans [29]; its expression lowers in the early bone marrow and thymocyte precursor cell compartments to increase again in the thymus following T cell maturation [30,31]. Peripheral blood T- and B-lymphocytes express modest levels of P-gp [32–35] that can be up-regulated upon lymphocyte activation in particular on CD4+ T cells.

P-gp expression can be measured by flow-cytometry using specific antibodies (CD243), and, its function, using rhodamine-123 (Rh-123) dye [14]. Rh-123 molecules enter living cells by passive effusion and are actively pumped out by P-gp. Thus, bigger is the loss of Rh-123 fluorescence higher is the function of the P-gp pumps. Because Rh-123 extrusion directly depends on P-gp, it can be blocked by verapamil, hydroxycloroquine, tacrolimus or cyclosporine that are P-gp inhibitors [36].

P-gp was first described to confer resistance to several chemotherapeutic drugs [37] or antagonizing caspase-3 dependent apoptosis in tumors [33,38]. More recently, P-gp function has been studied in multiple conditions in which patients develop resistance to therapy, for example P-gp expression has been correlated to the efficacy of highly active antiretroviral therapy (HAART) on HIV infection [39,40] and to the lack of response to CCS in autoimmune patients [41]. Although, genetic studies have shown that several polymorphisms on the *MDR-1* gene result in altered function of P-gp and increased susceptibility to develop colonic inflammatory diseases such as ulcerative colitis [42] no similar correlation has been exploited for other systemic autoimmune diseases.

The complex link between autoimmunity and P-gp function has been recently investigated by Ramesh *et al.* [3]. They showed that P-gp is a useful marker to distinguish a subgroup of pro-inflammatory human Th17 cells [43] from non-pathogenic Th17 cells; these lymphocytes produce both IL-17A, IL-17F, IL-22 and IFN-γ cytokines upon T cell receptor (TCR) stimulation and do not express IL-10 or other anti-inflammatory molecules. They also demonstrated that these Th17 cells expressing P-gp are refractory to several CCS and accumulate in the gut of Crohn’s disease patients.

## 3. Therapeutic Management and Drug Resistance in Systemic Lupus Erythematosus (SLE), Rheumatoid Arthritis (RA) and Psoriatic Arthritis (PsA)

The prototype of systemic autoimmune disease is SLE. SLE is a chronic autoimmune inflammatory disease of unknown etiology characterized by protean clinical manifestations that can involve different organs, such as kidney, heart, brain, skin and by the production of several autoantibodies against various self-molecules [44].

SLE emerges with a rate of 0.65/patient/year and has an increased mortality due to lupus activity, long lasting sequelae and heavy side effects caused by the present therapies, as compared to the general population [45,46]. Different immunosuppressant drugs can be implemented to control disease activity depending on the severity of and type of organ damage, particularly, nephritic *versus* neurological. Hydroxicloroquine (HYQ) and low dose of CCS are commonly effective in patients with mild SLE, whereas a mono or combined therapy of: azathioprine, methotrexate (MTX), mycophenolate mofetil and cyclophosphamide with high doses of CCS are used in active, severe or refractorySLE [47,48]. Cyclosporine (CsA) has been introduced in 1981 by Isenberg *et al.* [49] for patients with active SLE; 2.5–5 mg/kg/day may provide adequate disease control and behave as CCS-sparing drug [50]. CsA binds to cyclophilin with the synergistic action of calcium and suppresses activation of calcium-dependent phosphatase calcineurin, thus leading to the block of T cell activation and cytokine release, such as IL-2 [51]. CsA also represents a substrate and competitive inhibitor of the P-gp function [5].

Despite all the efforts, long term treatment of SLE still remains a major challenge for clinicians that have to face the constant fluctuation between remission and flares over time, drug unresponsiveness and side effects.

RA and PsA are chronic, inflammatory autoimmune diseases potentially leading to functional disability, deterioration in patients’ health-related quality of life and life expectancy [52].

There is strong evidence that the highest joint inflammation, observed in these patients, occurs in the first 2 years and that prompt treatment is able to slow down the progression of diseases reducing the inflammatory events associated with structural and functional joint impairment. Therapeutic weapons for RA and PsA are very similar and their number constantly increasing, as a consequence of a better knowledge of disease pathogenesis and of the advances in monoclonal antibody technology. Disease modifying anti rheumatic drugs (DMARDs), also called synthetic immunosuppressive agents, such as HYQ, sulfasalazine (SSA), leflunomide, MTX and CsA were, until 10 years ago, the most commonly used drugs to treat these patients. Nowadays they are often replaced or supported by the introduction of biological agents that target cytokines such as TNF-α, IL-6 or can inhibit T cell activation and B cell function. The gold standard therapeutic regimen for RA and PsA patients is represented by the early introduction of MTX plus low dose CCS, non responding patients can be treated with a combination of synthetic DMARDs or switched to a biological agent [53]. Although these treatment strategies have shown good efficacy, up to 40% of the patients may not benefit from the therapy because lack of efficacy, development of resistance [54] or treatment related adverse events [55]. Furthermore, this growing availability of therapeutic weapons is not supported by validate clinical/immunological predictive markers that can help the clinicians in the adoption of the best personal “targeted therapy”. CsA is a drug often used in PsA patients because it demonstrated to have good efficacy and high tropism for the skin [56,57]; however, it is important to note that also RA patients may benefit of a therapy using CsA specially in combination with MTX. In fact, a recent study by Migliore *et al.* [58] has shown that MTX + CsA was able to control disease activity and maintain clinical remission in RA patients more efficiently than therapy with MTX alone.

## 4. P-gp and Mutidrug Drug Resistance (MDR) in SLE, RA and PsA

The association between P-gp and MDR-1 has been extensively evaluated in oncology, whereas the studies on autoimmune diseases are scarcer and the few that do exist are mainly focused on patients with SLE and RA [54]. The first description, for a role of P-gp on autoimmunity, was reported in 1990 by a French group in which the authors described an overexpression of P-gp on the surface of peripheral lymphocytes of RA patients treated with CCS [59]. Later on, another group, Yudoh *et al.* in 1999 [60], showed an increased expression of P-gp on TH1 cells isolated from 22 RA patients not responding to SSA therapy. Hider *et al.* [61] found that MTX treatment in newly diagnosed RA patients resulted in the down regulation of P-gp expression on circulating mononuclear cells. In 2008 Tsujimura *et al.* [62] analysed P-gp expression on lymphocytes isolated either from healthy donors or from RA patients under therapy for at least 2 years and found that P-gp was overexpressed by lymphocytes from RA patients. Moreover, expression of P-gp positively correlated with disease activity and CCS treatment, whereas it reduced with MTX and with the use of infliximab, a chimeric monoclonal anti-TNF-α antibody. The same group reported similar results in 2010, this time analyzing 11 RA patients treated with the anti-TNF-α soluble receptor, etanercept [63]. Etanercept ensued a good disease control and was able to reduce P-gp expression on CD4+ T cells and on B cells (CD19+) in RA patients not responding to MTX as well as in two patients in which infliximab has failed. Agarwal *et al.* [64] evaluated P-gp expression in a group of 25 RA patients some of which were naïve and others were resistant to MTX therapy; the authors showed a significant positive correlation between high expression of P-gp and disease activity, whereas no association was found among high P-gp expression and unresponsiveness to MTX therapy. In contrast, Suzuki *et al.* [65] using the calcineurin inhibitor tacrolimus [66,67] (that works also as inhibitor of P-gp function) in 113 RA patients refractory to MTX or other DMARDs showed that 22 patients achieved good clinical responses correlated with a reduction of P-gp expression/function on peripheral lymphocytes. Interestingly Kis *et al.* in 2009 demonstrated that BCRP could play an important role in the development of resistance to leflunomide by using an ATPase assay, whereas neither leflunomide nor its active metabolite were capable of stimulating the basal activity of P-gp transporter, suggesting that the ABC proteins do not present DMARDs substrate profiles completely comparable [68]. Finally, we recently analyzed P-gp expression on three PsA patients with a secondary failure to MTX, before and after combined therapy with CsA and found that the introduction of CsA resulted in a good clinical outcome and a reduction on P-gp function on CD3+ T cells, particularly ascribed to the contribution of P-gp functional reduction on CD8+ T cells [69].

Several groups have invested considerable efforts on the study of the relationship between disease activity and P-gp expression levels/function in SLE patients [50]. In 2000, Llorente *et al.* [70] studied P-gp expression and function in the peripheral blood lymphocytes isolated from 30 SLE patients and showed that the mean percentage of lymphocytes with high P-gp activity was increased as compared to the healthy controls; moreover, lymphocytes from SLE patients in clinical remission had a lower P-gp activity than those with active disease. Similar results were obtained by the group of Tsujimura *et al.* in 2005 [71]. This group analyzed the expression of P-gp on peripheral lymphocytes from SLE and healthy controls and found significantly higher levels of P-gp on lymphocytes of 80 SLE patients with active disease than in normal controls. In addition, P-gp expression was increasing with the rise of disease activity and decreasing by immunosuppressive therapy. P-gp was expressed on both CD4+ and CD8+ T cells and strongly expressed by CD19+ B cells in patients with impaired response to CCS. The same group also demonstrated, first *in vitro* and then *in vivo*, that CsA is able to inhibit CCS excretion in freshly isolated lymphocytes from SLE patients and overcome CCS resistance once patients were treated with a combined therapy (CCS + CsA) [41]. More recently, Henmi *et al.* [72] confirmed that hyper function of P-gp on CD4+ T cells of 12 SLE patients correlated with a poor clinical response to CCS and Zhang *et al.* [73] corroborated the correlation between high P-gp expression in the peripheral blood lymphocytes and increased severity of SLE disease.

It is important to mention that P-gp expression/function has also been analyzed in other systemic autoimmune disorders such as idiopathic thrombocytopenic purpura (ITP) [74–76] and, more recently, inflammatory bowel diseases (IBD) [77]. Although they are few and involve only a small number of patients, the studies on IPT agree that resistance to CCS therapy is associated to hyper function of P-gp. As for IBD, the present data are often contradictory and need further elucidation. Indeed, a P-gp hyperexpression in circulating lymphocytes and epithelial cells of patients affected by Crohn’s disease and ulcerative colitis resistant to CCS has been described [78]. However, other authors did not find association between *MDR-1* polymorphism and response to therapy in a large cohort of pediatric patients with Crohn’s and ulcerative colitis [79].

## 5. Conclusions

ATP-binding cassette (ABC) transporters, including the P-gp transmembrane pump inequivocally play an important role in regulating intracellular drug concentrations. Several studies on patients with systemic autoimmune diseases in particular SLE, RA and PsA have demonstrated a significant correlation between P-gp expression/function, disease activity and the development of resistance to immunosuppressive therapy. Furthermore, P-gp inhibition/reduction by CsA or anti-TNF-α agents can overcome drug resistance, thus ameliorating patients outcome. In conclusion, although the matter deserves further studies, mainly as far as the new therapies are concerned, existing data already suggest that the measurement of P-gp expression/function could be a useful biomarker to evaluate drug resistance in these autoimmune conditions.
